# Editorial Comment: The Emerging Role of Stereotactic Ablative Radiotherapy for Primary Renal Cell Carcinoma: A Systematic Review and Meta-Analysis

**DOI:** 10.1590/S1677-5538.IBJU.2020.03.10

**Published:** 2020-02-20

**Authors:** RJM Correa, AV Louie, NG Zaorsky, EJ Lehrer, R Ellis, L Ponsky, Stênio de C. Zequi

**Affiliations:** 1 London Regional Cancer Program Department of Radiation Oncology LondonOntario Canada Department of Radiation Oncology, London Regional Cancer Program, London, Ontario, Canada; 2 University of Toronto Sunnybrook Health Sciences Centre Department of Radiation Oncology TorontoOntario Canada Department of Radiation Oncology, Sunnybrook Health Sciences Centre and the University of Toronto, Toronto, Ontario, Canada; 3 Penn State Cancer Institute Department of Radiation Oncology HersheyPA USA Department of Radiation Oncology, Penn State Cancer Institute, Hershey, PA, USA; 4 Icahn School of Medicine at Mount Sinai Department of Radiation Oncology New YorkNY USA Department of Radiation Oncology, Icahn School of Medicine at Mount Sinai, New York, NY, USA; 5 University Hospitals Seidman Cancer Center Case Comprehensive Cancer Center ClevelandOH USA University Hospitals Seidman Cancer Center, Case Comprehensive Cancer Center, Cleveland, OH, USA; 1 Fundação Prudente A. C. Camargo Cancer Center São PauloSP Brasil A. C. Camargo Cancer Center, Fundação Prudente, São Paulo, SP, Brasil

## COMMENT

Historically, urologists do not recommend external beam radiotherapy for patients with renal cell carcinoma. But the progresses of radiotherapy have permitted the use of a high-precision stereotactic ablative radiotherapy in hypofractioned scheduling. It seems a therapeutic alternative of for elderly and frail patients which are not surgical candidates. The rational is to offer a single session of 26Gy or a short curse of 40Gy, delivered only on five sessions, in an outpatient treatment. The endpoints must be tumor size reduction or no tumoral progression, being interesting for symptomatic candidates. The results are acceptable and side effects are tolerable. In face recent reports in the literature, Correa and several international key opinion leaders, published this metanalysis, that evaluated 26 selected studies. Reading this article, we can ourselves updated on this therapeutic approach, destinated for a subgroup of patients that usually has almost none options of treatment.

